# Invented spelling in English and pinyin in multilingual L1 and L2 Cantonese Chinese speaking children in Hong Kong

**DOI:** 10.3389/fpsyg.2022.1039461

**Published:** 2023-01-18

**Authors:** Yanling Zhou, Catherine McBride

**Affiliations:** ^1^Department of Early Childhood Education, The Education University of Hong Kong, Hong Kong SAR, China; ^2^Department of Human Development and Family Studies, Purdue University, West Lafayette, IN, United States

**Keywords:** invented pinyin spelling, invented English spelling, Cantonese phonological awareness skills, L1 Cantonese speaking children, L2 Cantonese speaking children

## Abstract

This research examined the relations among Cantonese phonological awareness, invented spelling in Pinyin (in Mandarin), and invented English spelling in 29 first language (L1) and 34 s language (L2) Cantonese-speaking second and third graders in Hong Kong. The purpose of this study was to understand how phonological awareness skills across languages are associated in multilinguals. We compared the phonological skills in the two groups (i.e., L1 and L2 Chinese speaking children) for the three official languages (i.e., Cantonese, Mandarin, and English) spoken in Hong Kong. The two groups did not differ on Cantonese phonological awareness, Mandarin Pinyin invented spelling, or English invented spelling, but the L1 group performed significantly better than the L2 group on Mandarin Pinyin tone skills, with non-verbal intelligence and grade level statistically controlled. In both groups, all three of the phonological sensitivity measures were significantly correlated with one another. With group, grade, and nonverbal IQ statistically controlled, only Mandarin Pinyin invented spelling but not Cantonese phonological awareness uniquely explained English invented spelling performance. In contrast, Pinyin invented spelling was uniquely explained by both English invented spelling and Cantonese phonological awareness skills. Results highlight some phonological transfer effects across languages.

## Introduction

How are phonological awareness skills across different languages interrelated for multi-literacy acquisition in multilingual speakers? Nowadays, more and more children acquire multiple languages from early on across the globe. Ample research has supported the phenomenon of phonological skill transfer for linguistically close language pairs (e.g., Durgunoğlu et al., 1993), as well as linguistically distinct bilingual learning (e.g., Yang et al., 2017). These results seem to support Cummins’ interdependence hypothesis of language transfer (Cummins, 1989), i.e., that phonological transfer is reciprocated among the languages within subject. However, most previous research has targeted L2 learners who acquire English or other alphabetic languages as an L2 (e.g., French, German, or Spanish). Despite a growing population of children whose first language is alphabetic and who acquire Chinese as an L2 (Zhou and McBride, 2018), little is understood about how such phonological transfer may affect such learners. In the context of Hong Kong, ethnic minority children represented by diverse alphabetic or Akshara speakers (e.g., Urdu, Hindi, Cebuano, etc.) lack a rich Chinese language and literacy environment at home. Yet they are expected, in the same way as their L1 Chinese speaking peers, to acquire Mandarin Chinese as a second or third language. Specifically, they use Cantonese, the home language of most families in Hong Kong, as the spoken language at school, but also learn Mandarin Chinese as an L3 in spoken and written forms, along with their Cantonese-speaking peers. In the present study, we aimed to understand how phonological skills in three languages are intercorrelated for L1 and L2 Chinese-speaking children. The three languages tested were Cantonese Chinese, Mandarin Chinese, and English. For one group of participants, all three of these languages were non-native languages, leading to intriguing questions in relation to overlap in phonological processes among them.

There were two primary focuses in this study: First, we examined how phonological awareness skills through a typical receptive phonological awareness task or an invented spelling task across the three languages, respectively, are correlated within the same children. Second, the study provides some insights into similarities and differences of such intercorrelations across L1 and L2 Chinese-speaking children.

### Phonological awareness skills and invented spelling in English

Invented spelling is a process by which a speller applies basic or partial conventional spelling rules for a sound representation that is auditorily perceived. As the term ‘invented spelling’ was coined originally or at least primarily for spelling in English, invented spelling in English captures children’s phonological awareness skills at the syllable and phoneme levels (e.g., Tangel and Blachman, 1992) and often serves as a gateway for reading and spelling, integrating phonological and orthographic knowledge (Invernizzi et al., 1994; McBride-Chang, 1998; Martins and Silva, 2006; Ouellette and Sénéchal, 2008; Ouellette et al., 2017; Treiman, 2018). Children’s invented spelling in English reveals the level of their understanding and creative mastery of phoneme-grapheme mapping rules (Gentry, 1982; Bear and Templeton, 1998) given the relative opaqueness of the English orthography as compared with other transparent alphabetic orthographies such as German or French. Therefore, early invented spelling is seen as a significant predictor of subsequent reading and writing abilities (Graham and Santangelo, 2014; Ouellette and Sénéchal, 2017). Invented spelling in English typically is regarded as an optimal tool for understanding children’s expressive phonological awareness skills because it requires children to demonstrate in writing explicitly how phonological representation is stored in their mind. It is also very useful for understanding lexical tone processing in Mandarin, since tonal notations are part of the Pinyin spelling process for this language. Invented spelling helps to indicate children’s progress and development in moving from experimentation of word representation in print to a later incorporation of orthographic accuracy (Richgels, 1995), rather than a pure memorization process of conventional spellings.

### Mandarin pinyin as a phonological facilitation in Chinese character acquisition

Similarly, invented Pinyin spelling is an effective tool to assess children’s explicit Chinese phonological awareness skills, and a strong facilitator for L1 Mandarin speaking children’s Chinese character reading skills (Lin et al., 2010; Ding et al., 2015, 2018) and L2 learners of Chinese (Ju et al., 2021; Zhang and Roberts, 2021). It captures syllable, phoneme, and tone knowledge (Lin et al., 2010). The pinyin system is a fabricated phonological system that was designed and implemented in the 1950s to facilitate early Mandarin Chinese literacy acquisition in China in order to address the issue of a relatively high rate of illiteracy (over 80%) in the country at the time (Zhou, 2013). The pinyin system is based on the Roman alphabetic system utilizing 26 letters representing consonants and vowels that map with Mandarin Chinese sounds. The morphosyllabic system of Chinese dictates that each character is an orthographic representation of a syllable; the syllable also represents a phonological unit. For majority of Chinese characters, there is a fixed onset-rime phonological structure which is usually attached to a lexical tone.

Children from all over China start to learn Pinyin in first grade at around the age of 6, for 10 weeks consecutively. In Mainland China, the school language is consistently Mandarin Chinese (Shu et al., 2003). That means that no matter what Chinese language or dialect children are speaking at home with their parents (e.g., Cantonese or Shanghainese, or Changsha Hua), students are required to speak and use Mandarin at school with their peers and teachers. In Hong Kong, the school language is usually Cantonese Chinese although the written language (vocabulary and grammar) that children need to acquire is in Mandarin Chinese (Cheung and Ng, 2003), since Mandarin Chinese is one of the three official languages and two literacies (Cantonese, English, and Mandarin) in Hong Kong.

Pinyin is one of several effective ways of learning Chinese characters (Packard, 1990; Chung, 2007; Everson, 2009; Poole and Sung, 2015; Zhang et al., 2017) together with other character learning methods such as writing focused (Guan et al., 2011) and character recognition focused approaches (Poole and Sung, 2015). However, In Hong Kong, children often begin to learn to read and to write Chinese characters and English words without any systematic phonological instruction at age 3.5 years as a cultural practice. Chinese characters are typically mapped to the Cantonese language for young children. Mandarin, another Chinese language, is also taught early in school in Hong Kong as a second language. However, pinyin, which is explicitly mapped only to Mandarin and not to Cantonese, is typically only taught as a part of the language arts subject beginning in primary school, several years after the initiation of Chinese literacy teaching, in Hong Kong.

### The similarities between the pinyin system and English spelling

Pinyin and English spelling share substantial commonalities and basic onset-rime mechanisms across their respective notation systems. There are 21 pinyin onsets, and some look the same as English consonants (e.g., b, p, f, and k). In contrast, a few pinyin onsets may look somewhat unusual in comparison to the English orthography (e.g., zh). Some sound representations are similar to those in English (e.g., d, t, s, and z), and some sound very different for the two languages (e.g., x, q, ch, and sh). There are 36 pinyin rimes, including rimes with a single vowel phoneme rime (e.g., a, o, and e), a double vowel rime (e.g., ai, ei, ao, and iong) or a rime that ends with a nasal sound (e.g., in, ing). Moreover, pinyin spelling includes a representation of lexical tone, with four basic and one neutral tones possible. Each Chinese character has its own tone attached to the syllable. Thus, each Chinese character represents a holistic syllable. To summarize, phonologically, each Chinese syllable comprises an onset and a rime, as well as a lexical tone, only; there are no consonant clusters in Chinese, as there are in English. The impact of English instruction on Pinyin spelling.

Previous research has demonstrated how acquiring English as a L2 strengthens native Chinese-speaking children’s pinyin skills (Chen et al., 2010; Ding et al., 2018). Ding et al. (2018) found that although invented Pinyin did not explain English conventional spelling in Mandarin-speaking children in Mainland China, their English invented spelling measure did explain unique variance in both their Chinese character writing and conventional English spelling performance. These researchers interpreted the English invented spelling findings as representing a linguistic transfer effect. Invented spelling indeed allows children the freedom to access their implicit knowledge of phonological representations of language or different languages in recoding sound patterns. Chen et al. (2010) found that learning English accelerates children’s pinyin skills. This research suggests a positive one-way transfer from English instruction to pinyin learning.

Up until now, research has primarily focused on native Chinese-speaking children’s pinyin knowledge. In contrast, very little is understood about the extent in which there is a transfer effect in multilingual children who are exposed to and learning Chinese as a second or third language (L2 or L3). In the present study, we aimed to obtain a comparative view of how phonological awareness skills in three languages might be associated among children living in Hong Kong. All of these children speak Cantonese as either L1 or L2. This focus can shed light on how multilingual children potentially organize their phonological system in the processes of multiple language and literacy acquisition. We examined phonological sensitivity in the form of Cantonese phonological awareness skills, as well as invented spelling in both pinyin (representing Mandarin, a prominent Chinese language) and English. As discussed below, invented spelling is a particularly sensitive way in which to capture phonological sensitivity (e.g., Mann et al., 1987; Lin et al., 2010). However, there is no such standard phonological written system available to, or at least common among, Cantonese-speaking children. Thus, our phonological sensitivity measure in Cantonese differed from the invented spelling measures in Mandarin and English, respectively.

### How does mastery of the pinyin system affect Chinese-English biliteracy learning?

Teachers sometimes worry that the two systems, which are written using similar notations (i.e., most of the Roman alphabet), could potentially interfere with one another in the learning process. Among scholars focused on native Chinese children’s learning, however, there has been a stronger argument that not only does pinyin learning not interfere with the acquisition of English learning (Lü, 2017), but it may actually have a positive effect on phonological awareness skills (e.g., McBride-Chang et al., 2004; Yin et al., 2011). For example, McBride-Chang et al. (2004) speculated that Beijing children, who typically perform significantly better on phonological skills at the syllable and phoneme levels than their Hong Kong counterparts, had an advantage precisely because of the Beijing children’s pinyin knowledge. Similarly, Cheung et al. (2001) compared phonological awareness skills between Cantonese speaking children in Guangzhou, China, and Hong Kong. They found a possible advantage resulting from pinyin training in the Guangzhou children on onset, rime, and coda analyses compared with their peers in Hong Kong. In the present study, we compared pinyin knowledge in two sets of L2 Mandarin Chinese learners. One group of children are L1 Cantonese Chinese speakers, and the other group represents ethnic minorities in Hong Kong who typically speak a variety of languages used in India, Pakistan, Nepal, and surrounding areas as their home languages. The Hong Kong Chinese group also speaks a tonal language as their first language (L1), but the ethnic minority group typically speaks a non-tonal language as their L1.

### Lexical tone as the Chinese phonological ‘twist’

Indeed, lexical tone sensitivity is fundamental for Chinese phonological awareness. Previous studies have highlighted the importance and close associations between tone awareness and both L1 (So and Siegel, 1997; Fu and Huang, 2000; Leong et al., 2005; Wang et al., 2005; Shu et al., 2008; Li et al., 2012; Tong et al., 2015) and L2 (Zhou and McBride, 2018) Chinese word recognition. Yin et al. (2011) argued that the stronger the pinyin knowledge, the stronger the tone awareness skills. Lexical tones in Mandarin Chinese are particularly difficult for learning Chinese as a foreign language. Yet tone sensitivity is essential for Chinese character learning given the many homophones of Chinese and meaning changes in Chinese with lexical tone changes. Zhou and McBride (2018) demonstrated that lexical tone was the weakest area of pinyin knowledge in L2 Chinese-speaking children, even when these children had been in an immersion Chinese-English classroom together with their L1 Chinese speaking peers for more than 4 years.

The present study compared native Cantonese speaking children with ethnic minority children learning Cantonese, Mandarin, and English all as second, or foreign languages. L2 Chinese speaking children in Hong Kong constitute approximately 6.4% of the Hong Kong children population (Census and Statistics Department, HKSAR, 2011). These students encounter great challenges in language and literacy development in Hong Kong public schools (Arat et al., 2016; Zhou et al., 2018). However, these children are expected to acquire Chinese and English language and literacy skills alongside their L1 Chinese speaking peers. We tested (a) the extent to which the two groups differed in phonological sensitivity across the three languages of Cantonese (*via* a phonological awareness task), Mandarin (*via* an invented Pinyin knowledge task), and English (*via* an invented spelling task) and (b) how these phonological sensitivity measures explained invented spelling in Pinyin and English, respectively.

## Materials and methods

### Participants

Second and third grade local primary school children were recruited for this study. There were 29 Cantonese Chinese-speaking children (L1) including 16 boys and 13 girls (Mean age = 93.4 months SD = 9.37 months) and 34 s language Chinese speaking ethnic minority children (L2) including 17 boys and 17 girls (Mean age = 94.0 months, SD = 5.35 months). The L1 Chinese speaking group comprised homogenous Cantonese speaking children with a Cantonese speaking home language and literacy background. Among the L2 group, the ethnicity backgrounds of the children included 14 Indian, 10 Nepalese and 10 Pakistani, respectively. For these children, Cantonese was the medium of instruction at school, English was taught as a foreign language, and Mandarin was learned and used for Chinese language arts classes in school.

### Procedure

Upon receiving parental consent, we worked with the school on scheduling testing sessions for individual children. Trained psychology undergraduate students and research assistants were employed for the data collection. They administered individual testing to the children at school or at after-school centers. Both L1 and L2 Chinese speaking children were divided into two groups, with some of the students beginning with Chinese tasks first and the remaining starting with English tasks for counterbalancing.

### Measures

#### Raven’s standard progressive matrices

Raven’s Colored Progressive Matrices B-C was used to evaluate the non-verbal reasoning of children (Raven et al., 1995). Twelve incomplete geometric figures were presented to the children one by one. For each question, children were asked to choose one pattern from among 6–8 choices in order to make the figure complete. One point was given to each correct answer and the total score for this test was 12.

#### Chinese word reading

This test was composed of 211-items. Twenty-seven of them were single Chinese characters, 34 of them were two-character Chinese words which were frequently used, and the remaining 150 items were two-character Chinese words from the subset of The Hong Kong Test of Specific Learning Difficulties in Reading and Writing (Ho et al., 2000). Previous research has also adopted a similar measure to access the Chinese word reading ability of children (e.g., McBride-Chang et al., 2003; Zhou and McBride, 2018). Despite the number of characters for each item, one point was given to children who answered correctly for an item. Thus, the total points possible for this task was 211.

#### English word reading

Previous research studies had developed a task for examining the English reading ability of children (Tong and McBride-Chang, 2010; Zhou and McBride, 2018). Due to a previous observation of some likelihood that L2 Chinese speaking children may have higher proficiency in English, for the present study, we expanded the word list based on a previous measure adopted (Zhou and McBride, 2018). According to the children’s reading ability, difficulties of word meaning and phoneme combinations, sixty-three words were selected to assess the English word reading ability of children. One point was given for each correct pronunciation, so the maximum points for this test was 63. The test was terminated immediately when the children mispronounced four consecutive words.

#### Chinese vocabulary knowledge

The test was divided into three subtasks to assess the knowledge of receptive vocabulary, expressive vocabulary, and vocabulary definitions of children. All the items in this section were consistent with the children’s age of acquisition and ordered according to ascending difficulty. We tested vocabulary knowledge orally only, as a point of comparison to understand oral language skills. For receptive vocabulary, there were 21 items and the experimenter presented four pictures to children for each item. Children were required to choose a picture from among the four choices which best matched the meaning of the Chinese word that was verbally presented by the experimenter in Cantonese. One point was given for each correct response. For expressive vocabulary, there were 23 items and all the pictures were obtained from the Peabody Picture Vocabulary Test-Third Edition (PPVT-III; Dunn and Dunn, 1997). Children were required to name the object or situation in Cantonese corresponding to the picture shown. One point was given for each correct response. For vocabulary definitions, there were 27 items. The experimenter spoke a word in Cantonese to children and children were required to provide the definition of this word. Children were given 0–2 points per item based on the precision of their answer. If children answered five consecutive questions incorrectly in one of the subtasks, the experimenter stopped and returned to the remaining subtasks of the test. The total score for this test was 98.

#### English vocabulary knowledge

Similar to the procedure described above for Chinese vocabulary knowledge testing, tasks of receptive vocabulary, expressive vocabulary and vocabulary definitions were included in the test of English vocabulary knowledge. There were 21, 23, and 26 items in each subtask, respectively. The words for this test were extracted from the PPVT-III (Dunn and Dunn, 1997). The order of the words was based on the familiarity and difficulty levels. The English vocabulary knowledge test adopted the same scoring criteria that were used for Chinese vocabulary knowledge. The total score for this test was 96.

#### Cantonese phonological awareness

Items tapping syllable deletion and onset deletion were the measures used to assess children’s Cantonese phonological awareness. Previous studies (McBride-Chang et al., 2005; Cheung et al., 2009) had adopted these two subtasks to assess the Cantonese phonological awareness of children. All the stimuli in this test were presented verbally. The syllable deletion task consisted of 29 items, and 15 of them were three-syllable real words while the remaining were pseudowords. Children were asked to drop one of the syllables of a word and then say what was left after removing that syllable. For instance, children were asked to say *漢堡包* /hon3/ /bou2/ /baau1/ without/baau1/. So, children needed to say*漢堡* /hon3/ /bou2/ in order to receive one point. The onset deletion task consisted of 22 items, and ten of them were one-syllable real words while the remaining were one-syllable pseudowords. Children were asked to delete the first consonant of the word in each item and say the word without the initial sound. For instance, children needed to reply with /i1/ when they were told to remove the consonant of *詩* /si1/. One point was given for each correct response. Children started at the level consistent with their own grade level, and both subtasks were terminated when they reached the limit of the stopping rule. The total score for this test was 51.

#### Pinyin invented spelling (mandarin)

A previous study had developed this task for examining the Mandarin Pinyin invented spelling of children (Lin et al., 2010). For the present study, we also adopted a similar measure and scoring scheme. There were ten double syllable real pinyin words in the test and children were required to write down the Pinyin representation of the words that were verbally presented by the experimenter. Please see the Appendix for the list. As the verbally presented pinyin words (e.g., táng dài) were presented in the absence of a given context or sentence, these were conceptualized as appropriate for this task, since the items could be recognized as homophones of different words. With this approach, the Pinyin items were not too novel, reducing the cognitive load in processing unfamiliar sound combinations, but the items were also challenging. Relatively few words were written 100% correctly. For each pinyin syllable a child wrote down, we adopted scales for the onset (0–4 points), rime (0–4 points), lexical tones (0–3 points) and the order of Pinyin (0–1point). For scales for onset and rime, the scores were given based on the closeness of the written pinyin letter to the targeted pinyin. For example, 0 points were given if nothing was written; 1 point was given if a random letter was written down; 2 points were given if the component included a letter that was exactly or close to the target onset or rime, but had multiple irrelevant letters; 3 points were given if the component had a phonologically similar letter but not the accurate pinyin letter; 4 points were given if the component had exactly the targeted pinyin letter or letters. For the lexical tone evaluation, the correct tone was coded as 3 points and incorrect tones were coded as 1, but if nothing was written to indicate the tone, then 0 points were given. For each pinyin syllable, the maximum score was 12, so across the 20 Pinyin syllables altogether, the total possible score was 240. Based on this coding system, two variables were created, namely, (1) invented pinyin spelling in total that was inclusive of lexical tone score, and (2) the invented pinyin tone score alone.

#### English invented spelling

Unlike the English word dictation task, 8 pseudo words with single syllables were constructed, some of them containing digraphs and double consonants, for this task. Children were required to write the pseudo words when they heard the sounds. The scoring procedure was adopted from two previous studies on a 0–5 point scale (Mann et al., 1987; Tangel and Blachman, 1992). Children received 0 points when they did not make any response or answered the question randomly. One point and two points were allotted for each item on which children only correctly wrote the phonetically related letter(s) and initial letter, respectively; three points were given for items written in a pre-conventional manner with at least one phoneme correct; four points were given for items for which all phonemes were correctly written but in a pre-conventional manner; and five points were given for each item for which the pseudo word was correct. Thus, the total score for this test was 40.

## Data analysis

A two-step statistical analysis was carried out. First, MANCOVAs were employed to determine whether there were differences between L1 and L2 groups on Chinese and English word reading related tasks. The result of Box’s test did not allow us to reject the null hypothesis of equal covariance matrices for the MANCOVA tests. We then ran a hierarchical regression to test how phonological awareness skills in the other two languages predicted English invented spelling and Pinyin invented spelling for L1 and L2 groups similarly and differently. We computed dummy variables setting L1 as the reference group and L2 as the comparison group in order to examine the similarities and differences within the same model.

## Results

Multiple ANCOVAs were carried out to examine the group differences on Chinese and English word reading related measures after controlling for non-verbal intelligence and grade. There was a significant major group effect, *F*(1,8) = 36.6, *p* < 0.001. As found previously (e.g., Zhou et al., 2018; Zhou and McBride, 2018), as the test of pairwise comparisons shows in Table 1, L2 Chinese speaking children demonstrated a clear advantage in English. They performed significantly better on English word reading and vocabulary knowledge than did the L1 Chinese speaking children. L1 Chinese speaking children demonstrated an advantage in Chinese word reading and vocabulary knowledge, compared with L2 Chinese speaking children. However, the two groups performed similarly on all phonological tasks across the three languages, including Cantonese phonological awareness skills and overall invented Mandarin Pinyin skills; the only difference was Chinese lexical tones. L1 Chinese speaking children performed significantly better than the L2 children in Mandarin tone awareness skills. Therefore, it appears that lexical tone in L2 Chinese speaking children who learn Chinese as a L2 is the weakest for acquiring Chinese phonological awareness skills, echoing previous findings for L2 Chinese speaking children whose first languages are nontonal (Zhou and McBride, 2018; Table 2).

**Table 1:** Descriptive statistics of L2 Chinese speaking and L1 Chinese speaking students’ performance on Chinese and English related tasks after controlling for nonverbal intelligence and grades.

Variables	L2 (*N* = 34)		L1 (*N* = 29)		Pairwise comparison mean differences (L2–L1)	Reliability coefficiency
	Mean	SD	Mean	SD		
CWR	26.09	19.12	128.52	33.06	−95.62^***^	0.99
EWR	39.77	14.83	29.46	17.93	11.50^*^	0.98
CV	17.28	9.55	53.03	13.31	−33.79^***^	0.97
EV	46.39	10.13	34.97	18.76	13.44^**^	0.95
CPAS	28.82	11.01	35.69	11.10	−4.63	0.97
MPIST	30.88	12.73	46.67	10.10	−9.29^**^	0.96
MPIS	123.21	42.43	143.37	54.69	−4.59	0.96
EIS	18.06	5.67	17.59	6.70	1.92	0.78

^*^p < 0.05, ^**^p < 0.01, ^***^p < 0.001.

**Table 2:** Partial correlation between all the literacy related measures in L2 Chinese speaking and L1 Chinese speaking children after controlling for nonverbal intelligence and grade (L2_Left bottom up triangle/ L1_ right corner down triangle).

Variables	1	2	3	4	5	6	7	8
1. Chinese Word Reading	–	−0.24	0.70^***^	−0.18	−0.07	0.26	−0.04	−0.17
2. English World Reading	0.54^**^	**–**	−0.21	0.80^***^	0.35	0.32	0.54^**^	0.71^***^
3. Chinese Vocabulary	0.59^**^	0.47^**^	**–**	−0.21	0.14	0.27	0.12	−0.08
4. English Vocabulary	0.22	0.53^**^	0.30	**–**	0.42^*^	0.19	0.53^**^	0.64^***^
5. Cantonese PA	0.54^**^	0.52^**^	0.41^*^	0.09	**–**	0.30	0.55^**^	0.40^*^
6. M-Pinyin Invented Spelling _Tone	0.12	0.35^*^	0.11	0.03	0.10	**–**	0.70^***^	0.32
7. M-Pinyin Invented Spelling Total	0.38^*^	0.66^***^	0.26	0.39	0.45^*^	0.60^***^	**–**	0.48^**^
8. English Invented Spelling	0.41^*^	0.74^***^	0.45^***^	0.28	0.51^**^	0.33	0.55^***^	**–**

The correlations shown in Table 3 indicate that after statistically controlling for grade and non-verbal intelligence, Chinese word reading was significantly associated with English word reading skills (*r* = 0.54, *p* < 0.01), Cantonese phonological awareness skills (*r* = 0.54, *p* < 0.01), Pinyin invented spelling skills (*r* = 0.38, *p* < 0.05), and English invented spelling skills (*r* = 0.41, *p* < 0.05) for L2 Chinese speaking children. However, L1 Chinese speaking children’s Chinese word reading skills was negatively associated with their English word reading (*r* = −0.24, p < 0.05), and unassociated with phonological sensitivity in all three languages. English word reading was also significantly associated with Mandarin Pinyin invented spelling skills (*r* = 0.54, *p* < 0.01) and English invented spelling skills (*r* = 0.71, *p* < 0.01), but not with Cantonese phonological awareness skills in the L1 Chinese-speaking group.

**Table 3:** Summary of Hierarchical regression predicting English Invented Spelling of L1 and L2 Chinese speaking children.

Variables		Change	*F* change	*β*	*t*
Step 1	0.20	0.20	4.84^**^		
Group				−0.18	−1.35
Ravens				0.45	3.50^***^
Grade				0.20	1.63
Step 2	0.37	0.18	7.93^***^		
Group				−0.43	−1.29
Ravens				0.26	2.09^*^
Grade				0.05	0.47
Cantonese PA				0.55	3.11^*^
DummyG_PA				−0.15	0.64
Step 3	0.49	0.12	6.17^**^		
Group				−0.14	0.39
Ravens				0.20	1.68
Grade				0.14	1.20
Cantonese PA				0.18	1.31
DummyG_PA				−0.01	−0.02
MPinyin Invent Total				0.39	2.25^*^
DummyG_MPinyin Invent				0.12	0.32

Cantonese PA = Cantonese Phonological awareness; DummyG_ = L1 as reference group, 0, L2 as comparison group, 1 ^*^p < 0.05, ^**^p < 0.01, ^***^p < 0.001.

For the L2 group, English word reading skills were significantly associated with phonological skills in all three languages [i.e., Mandarin Pinyin invented spelling skills (*r* = 0.66, *p* < 0.01), English invented spelling skills (*r* = 0.74, *p* < 0.01) and Cantonese phonological awareness skills (*r* = 0.52, *p* < 0.01)].

Phonological skills in the three languages were significantly intercorrelated in both the L1 and L2 groups. English invented spelling skills were significantly associated with Cantonese phonological awareness skills (L1: *r* = 0.40, *p* < 0.05; L2: *r* = 0.51, *p* < 0.01) and Mandarin Pinyin invented spelling skills (L1: *r* = 0.48, *p* < 0.01; L2: *r* = 0.55, *p* < 0.01). Cantonese phonological awareness skills were significantly associated with Mandarin Pinyin invented spelling skills (L1: *r* = 0.55, *p* < 0.01; L2: *r* = 0.45, *p* < 0.05).

Hierarchical regression analyses were carried out to examine how similarly and differently phonological skills in the three languages predicted the English invented spelling skills and Mandarin Pinyin Invented spelling skills for the L1 and L2 groups. We computed a dummy variable and set L1 as the reference group and L2 as the comparison group given the above-mentioned correlational differences in the groups. As shown in Table 3, the regression models revealed that for English invented spelling skills, at step 2 only the Cantonese phonological awareness skills task was a significant predictor for English invented spelling skills for both groups without considering Mandarin Pinyin invented spelling, suggesting a moderate transfer effect. After introducing Mandarin Pinyin invented spelling skills and the dummy variable of Mandarin Pinyin invented spelling skills to the regression model at step 3, only the Pinyin invented spelling skills task was a significant predictor for English invented spelling skills for both groups. For Mandarin Pinyin invented spelling skills, Cantonese phonological awareness skill was a significant predictor for both groups. English invented spelling skills and its dummy variable were entered at Stage three of the regression. Both Cantonese phonological awareness skills and English invented spelling skills were significant predictors of Mandarin Pinyin invented spelling skills, which also highlights transfer of phonological awareness skills of both Cantonese and English in learning Mandarin Pinyin skills. The two groups did not differ on how phonological awareness skills in the three languages predicted English invented spelling skills and Mandarin Pinyin invented spelling skills, respectively (Table 4).

**Table 4:** Summary of hierarchical regression predicting Mandarin Pinyin Invented Spelling of L1 and L2 Chinese speaking children.

Variables		Change	*F* change	*β*	*t*
Step 1	0.15	0.15	3.41‑		
Group				0.02	0.16
Ravens				0.37	2.82^**^
Grade				−0.03	−0.21
Step 2	0.37	0.23	10.26^***^		
Group				−0.58	−1.73
Ravens				0.15	1.19
Grade				−0.16	−1.43
Cantonese PA				0.72	4.10^***^
DummyG_PA				−0.51	−1.55
Step 3	0.49	0.11	6.11^**^		
Group				−0.41	1.18
Ravens				0.04	0.31
Grade				−0.19	−1.75
Cantonese PA				0.48	2.51^*^
DummyG_PA				−0.42	−1.14
English Invented Spelling				0.44	2.54^*^
DummyG_EIS				−0.03	−0.09

Cantonese PA = Cantonese Phonological awareness; DummyG_ = L1 as reference group, 0, L2 as comparison group 1; EIS = English Invented Spelling. ^*^p < 0.05, ^**^p < 0.01, ^***^p < 0.001.

## Discussion

The present study extended our understanding of the significant correlations of phonological skills in three different languages (Mandarin, Cantonese, English) in multilingual children across at least at two levels: First, the significant positive correlations across measures of phonological sensitivity suggest a kind of shared phonological mechanism that supports languages that are closely related to each other (i.e., Mandarin and Cantonese) and the languages that are distinct from each other (i.e., Mandarin/ Cantonese Chinese and English). Our results showed that strong phonological skills in oral Cantonese Chinese were associated with strong phonological skills in Mandarin and English, respectively. Pinyin knowledge as an acquired alphabetic system that is intended to support Chinese character recognition has a positive ‘side effect’ on English phonological awareness skill. In turn, strong awareness of English phono-grapheme rules as indicated *via* invented English spelling was positively associated with invented Pinyin spelling.

Second, this pattern was demonstrated not only among L1 Chinese speaking children but also for L2 learners. Although L1 Chinese speaking children demonstrated a clear advantage in pinyin spelling, the patterns for both groups demonstrated that at least for older children, even for children whose first language is different from these two languages, the pinyin and English invented spellings are two distinct alphabetic notation systems, though they are relatively strongly correlated. This is perhaps particularly important for L2 language Chinese young learners whose first languages are neither Chinese nor English. Pinyin learning can perhaps serve as a bridge for them in acquiring Mandarin Chinese phonological awareness skills. Similarly, for Cantonese speaking children who learn Mandarin Chinese as a written language, not only can pinyin knowledge possibly influence their English spelling skills, and, hence, their phonological awareness skills, but it also relates significantly to their L1 Cantonese phonological skills. Given that there is no official agreed upon Cantonese phonetic system taught at school, Mandarin pinyin skills can potentially have a positive effect for Chinese literacy acquisition. One of the limitations of the present study is that the examination of Cantonese phonological awareness skills was done verbally and Cantonese lexical tones were not included in the present study due to a lack of a coherent system that is explicitly introduced in the education system in Hong Kong. Future studies could explore phonological awareness skills at different levels including lexical tones, using auditory tasks (rather than written tasks) only for the three languages.

A side note from this study that should be highlighted is the fact that, although the total phonological sensitivity measures in Cantonese, Mandarin, and English were all moderately and significantly associated with reading in English for both L1 and L2 Chinese speaking children, none of these were associated with Chinese reading for L1; however, for L2 Chinese speaking subjects, the significant associations with Chinese reading and vocabulary remained moderate to strong. A lack of associations for Chinese word reading in L1 highlights the differing demands of literacy acquisition for Chinese as compared to English (Chung and Ho, 2010). English reading relies substantially on phonology. For L2 populations, similar to the results in a previous study (Zhou and McBride, 2018), the reliance on phonological awareness skills may be the major strategy, though not sufficient enough, to acquire Chinese literacy. In the Hong Kong context, where reading is not taught using a phonological coding system, as it is for the rest of Mainland China, phonological skills may be required only in a very limited way in order to master word recognition.

Moreover, Mandarin lexical tones are clearly the most difficult aspect of Mandarin pinyin spelling to acquire for ethnic minority L2 Chinese speaking children when compared with L1 Chinese speaking children in Hong Kong. However, the comparable results between the two groups that were found for Cantonese phonological awareness, English invented spelling skills, and Mandarin pinyin, but not in Chinese word reading and vocabulary, were consistent with previous findings (Zhou and McBride, 2018) demonstrating that L1 and L2 Chinese speaking children did not differ on their phonological awareness skills in Chinese and English but did differ significantly on Chinese word reading and vocabulary. Lexical tones, which are absent in many alphabetic languages, are a critical indicator of Chinese vocabulary and word learning (Liu et al., 2010; Zhou and McBride, 2018). The complete absence of lexical tones in English and many other languages renders them more difficult for L2 Chinese speaking children to process, remember, and produce when compared with their L1 peers. The weakness in Mandarin lexical tones in the L2 children in the present study is also at least partly likely attributable to a lack of exposure and language experience in Mandarin Chinese as the school and peer language is mainly Cantonese. Mastering lexical tone may reflect overall oral language skills, which serve as a strong foundation for acquiring Chinese word reading (Zhou and McBride, 2018).

The present study aimed to explore possible transfer effects of phonological skills in English, Mandarin, and Cantonese in L1 and L2 Cantonese speaking children. In this study the identified transfer effect, both as a learning mechanism (Thorndike, 1913; Brown and Kane, 1988) and a linguistic effect (Corder, 1983; Cummins, 1989) enhanced our two understandings about phonological transfer. First, transfer evolves from implicit knowledge of one’s native language to explicit knowledge of the L2 or L3 language (Corder, 1983); in the present study, this transfer effect emerged for both L1 and L2 Cantonese speaking children, even though English is a distant language from Mandarin and Cantonese, and words in the latter two languages have simpler phonological structures (i.e., consonant and vowel or onset and rime) than that for English words. Thus, the nature of combining speech sounds together to form individual words applies across all three languages in oral form as well as in the form of invented spellings of English and Mandarin.

Despite the relatively small sample size given various difficulties in recruiting L2 Cantonese learners at this grade level, we were able to identify positive correlations between spelling and phonological awareness in three languages in L1 and L2 Chinese speaking students in Hong Kong. The present study demonstrated that acquisition of Mandarin through the pinyin system as another language for both L1 and ethnic minority children is positively associated with spelling and phonological awareness skills in other languages among multilingual Hong Kong children. Future studies should consider further examining phonological awareness skills using oral tasks across all three languages within this multilingual population. The patterns identified through the present study, which shed light on the transferability of phonological sensitivity in multilingual children, should be further explored in future work.

## Data availability statement

The raw data supporting the conclusions of this article will be made available by the authors, without undue reservation.

## Ethics statement

The studies involving human participants were reviewed and approved by The Education University of Hong Kong. Written informed consent to participate in this study was provided by the participants' legal guardian/next of kin.

## Author contributions

YZ analyzed the data and drafted the manuscript. CM had the data collected. All authors contributed to the article and approved the submitted version.

## Funding

This research project was funded by the Public Policy Research Funding Scheme (Project Number: 2013.A4.001.13A) from the Central Policy Unit of the Hong Kong Special Administrative Region Government and by the General Research Fund of the Hong Kong Special Administrative Region Research Grants Council (Project Number: 14600818 and 18611218).

## Conflict of interest

The authors declare that the research was conducted in the absence of any commercial or financial relationships that could be construed as a potential conflict of interest.

## Publisher’s note

All claims expressed in this article are solely those of the authors and do not necessarily represent those of their affiliated organizations, or those of the publisher, the editors and the reviewers. Any product that may be evaluated in this article, or claim that may be made by its manufacturer, is not guaranteed or endorsed by the publisher.

## Appendix I: Mandarin pinyin invented spelling list

1. bú yào
2. táng dǎi
3. bǐng gān
4. méi yǒu
5. sān gúo
6. zhī chí
7. qiǎo miào
8. lǜ sè
9. rì chū
10. jiàng xuě
