# Supplementary figures and images for: Invented spelling in English and pinyin in multilingual L1 and L2 Cantonese Chinese speaking children in Hong Kong
Source: Front Psychol. 2023 Jan 18;13:1039461. doi: 10.3389/fpsyg.2022.1039461 (PMC9889635; doi:10.3389/fpsyg.2022.1039461)

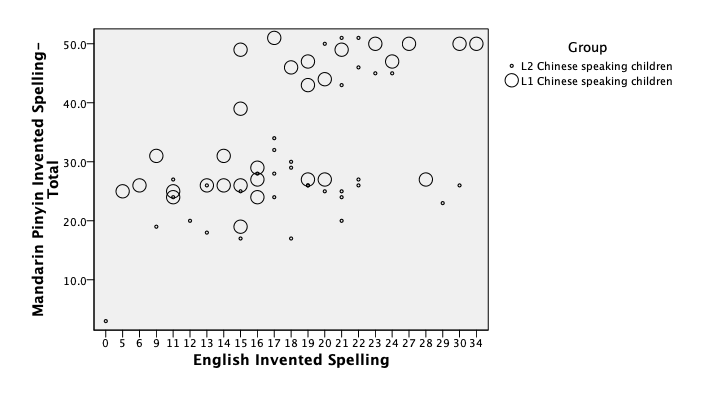


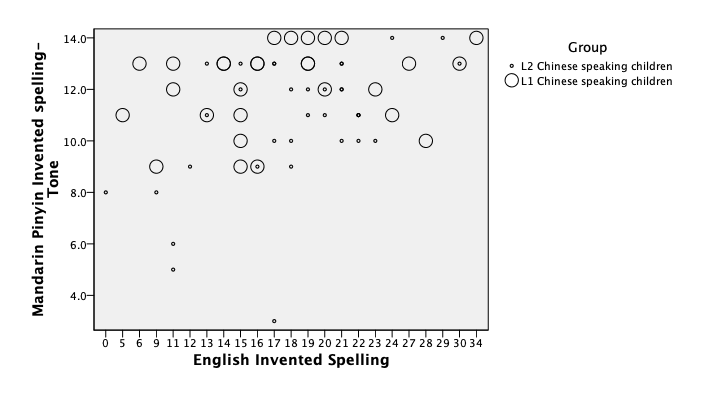

Supplement: Supplementary file 1 [file Data_Sheet_1.docx]
